# Primary synovial sarcoma of the parapharyngeal space: a clinicopathologic study of five cases

**DOI:** 10.1186/1477-7819-10-158

**Published:** 2012-08-03

**Authors:** Ming Zhu, Jun Li, Ke-Jing Wang, Jin-Biao Shang

**Affiliations:** 1 Department of Head and Neck, Zhejiang Cancer Hospital, Hangzhou, 310021, Peoples Republic of China; 2 Department of Pathology, The First Affiliated Hospital, College of Medicine, Zhejiang University, Hangzhou, 310003, Peoples Republic of China

**Keywords:** Synovial sarcoma, Parapharyngeal space, Molecular pathology, *SYT–SSX* gene fusion, Surgery, Prognostic factors

## Abstract

We report five cases of primary synovial sarcomas arising in the parapharyngeal space. The patients were all men with a median age of *35* years (range *22* to *41* years). The tumors were non-encapsulated solid masses ranging from *2.0* to *6.6* cm in size. Histologically, three cases were biphasic subtype, and the other two cases were monophasic subtype. Immunohistochemically, the tumor cells were strongly positive for bcl-2 and CD99, partly positive for CK and EMA, and negative for CD117, CD34, SMA and desmin in all five cases. S-100 protein was detected in one case. The presence of an *SYT–SSX1* and/or *SYT-SSX2* gene fusion resulting from *t(X;18)* was demonstrated from paraffin blocks by reverse transcriptase polymerase chain reaction in five cases. All five patients received tumor radical excision and postoperative radiotherapy, and two patients with pulmonary metastasis received additional chemotherapy. Follow-up data revealed that two patients with tumor size <*5* cm were alive without disease for *54* and *57* months, one patient with tumor size <*5* cm was alive with pulmonary metastasis for 78 months, and two patients with tumor size >*5* cm died of disease *26* and *37* months after the diagnosis, respectively. Primary parapharyngeal synovial sarcoma is a rare variant that occurs more frequently in males than females. Accurate diagnosis depends on morphologic and immunohistochemical examination and proper molecular analysis. The prognosis is relatively good in those patients whose tumor size is less than *5* cm.

## Background

Parapharyngeal space (PPS) tumors comprise approximately *0.5%* of head and neck tumors [1]. Synovial sarcoma (SS) of the head and neck is rare, accounting for only less than *10%* of all head and neck soft tissue sarcomas [2]. Very few reports of primary parapharyngeal synovial sarcoma (PPSS) have been published, and most of these reports have been based on histological and immunocytochemical examination. In this study, we describe five cases of PPSS diagnosed with the assistance of histological,immunocytochemistry and molecular pathology.

## Case presentation

Among this group, four cases of PPSS were from Zhejiang Cancer Hospital and the other was from the First Affiliated Hospital, College of Medicine, Zhejiang University.

All five patients were men with a median age of *35* years (range *22* to *41* years). On physical examination there was a mass arising from the intraoral, neck or pharyngeal space in five patients causing dysphagia (two of five) and pain (one of five) for *1* to *12* months. No patients had a previous history of sarcoma or radiation at the tumor site.

Although fine needle aspiration biopsy (FNA) biopsies were performed in two patients, pathologists interpreted these cytological specimens as a parotid carcinoma or neurogenic neoplasm. In one patient, an intraoral open biopsy was carried out and achieved correct diagnosis of PPSS.

All five patients were treated with surgical extirpation of the tumor. Transcervical and transmandibular approaches were performed in four patients and one patient, respectively. Complete gross examination was available for all five tumors, and the surgical margins were all negative in the excision specimens. No lymph node metastases in the surgical specimen of regional or total neck were found in two patients.

**Figure 1:**
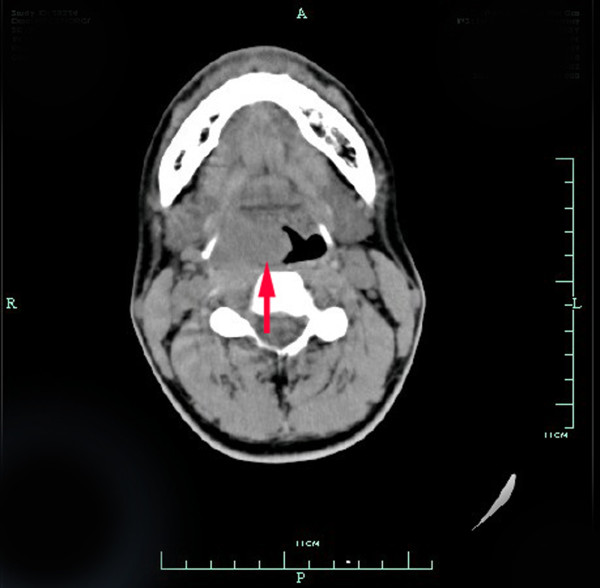
**CT scan showing a *3.0* -cm mass of the parapharyngeal space in Case *2* **.

**Figure 2:**
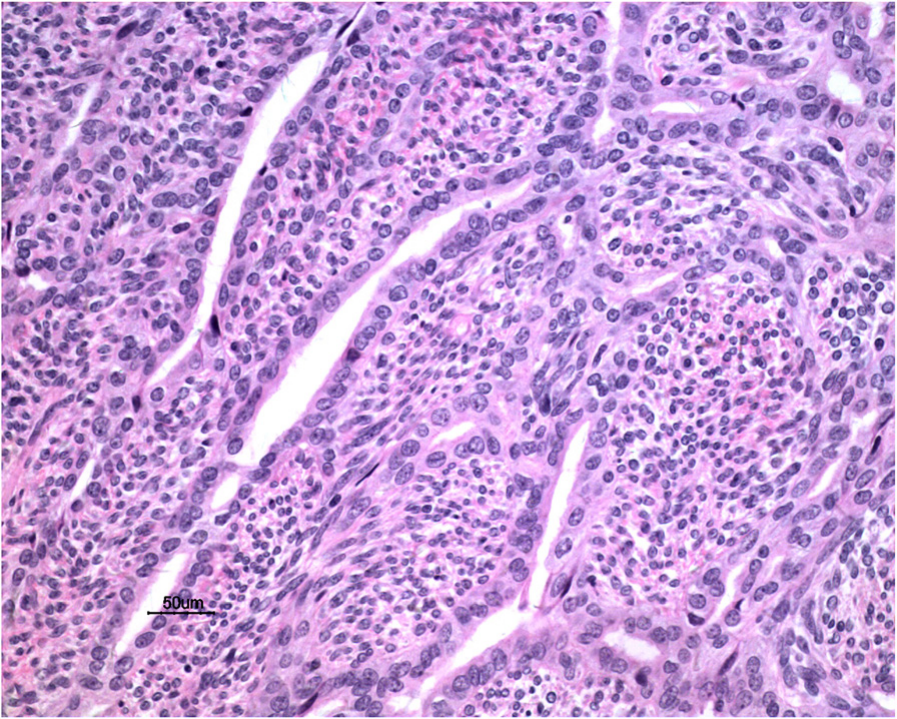
**Histological feature revealed biphasic pattern composed of spindle-shaped cells and glandular spaces in Case *3* (H&E, *20* ×)**.

**Figure 3:**
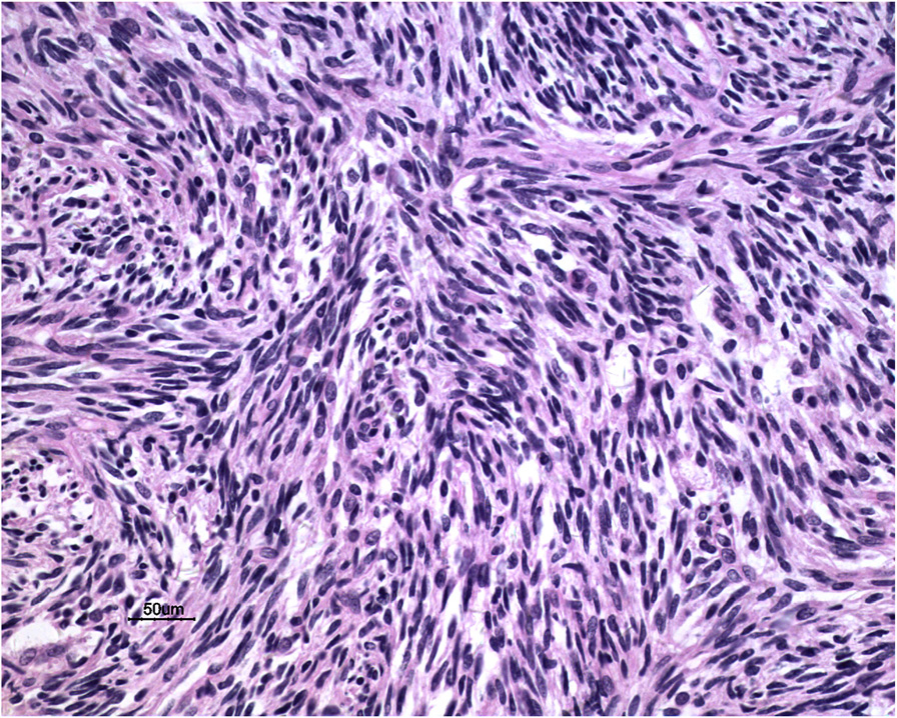
**Case 1: the tumor had a monophasic appearance composed of spindle cells forming interlacing fascicles (H&E, *20* ×)**.

**Figure 4:**
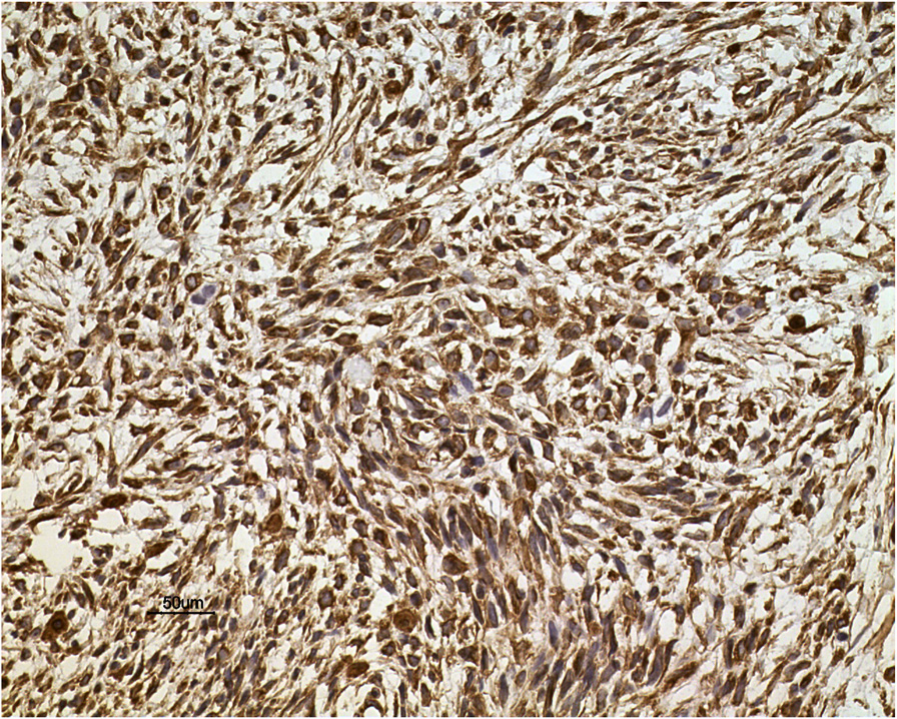
**By immunohistochemistry, the tumor cells of Case *5* were strongly positive for vimentin (DAB, *20* ×)**.

**Figure 5:**
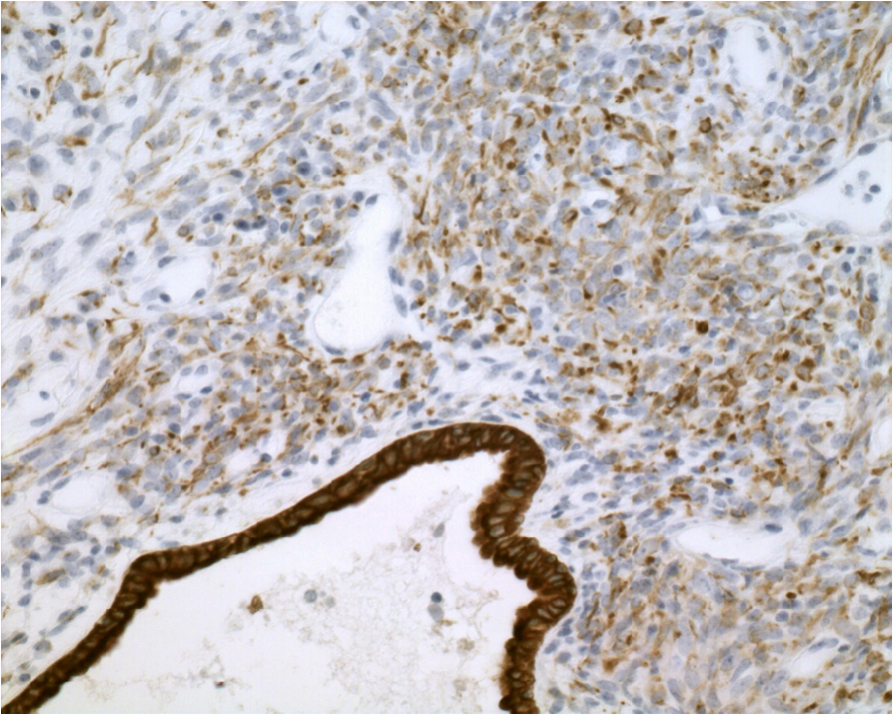
**Case *4* : CK was positive in the spindle cells and the glandular part (DAB, *20* ×)**.

Macroscopically, the tumors were gray-white, elastically soft, areas of necrosis and hemorrhaging, non-encapsulated solid masses, ranging in size from *2.0* to *6.6* cm (Figure 1). Histologically, the tumor in three cases presented a biphasic subtype composed of spindle-shaped cells and epithelial cells (Figure 2). Architectural formations characteristic of epithelial differentiation, including acinar-like structures, tubules (glands) and flat sheets, all dissociated from spindle cells. The other two cases had a monophasic appearance, composed mainly of uniform spindle cells forming interlacing fascicles (Figure 3). Portions of the tumor showed a hemangiopericytomatous pattern consisting of polygonalcells arranged around dilated, thin-walled blood vessels. By immunohistochemistry, both epithelial and spindle tumor cells were strongly positive for bcl-2 and CD99, and the spindle cells were diffusely expressed vimentin (Figure 4) in all five cases. CK and EMA were partly positive in the spindle cells and diffusely positive in the glandular part (Figure 5) of three biphasic cases, as well as focally positive in the spindle cells of the other two monophasic cases. S-100 protein was focally positive in one monophasic case. The tumor cells of the five cases were negative for SMA, desmin, CD117 and CD34.

**Figure 6:**
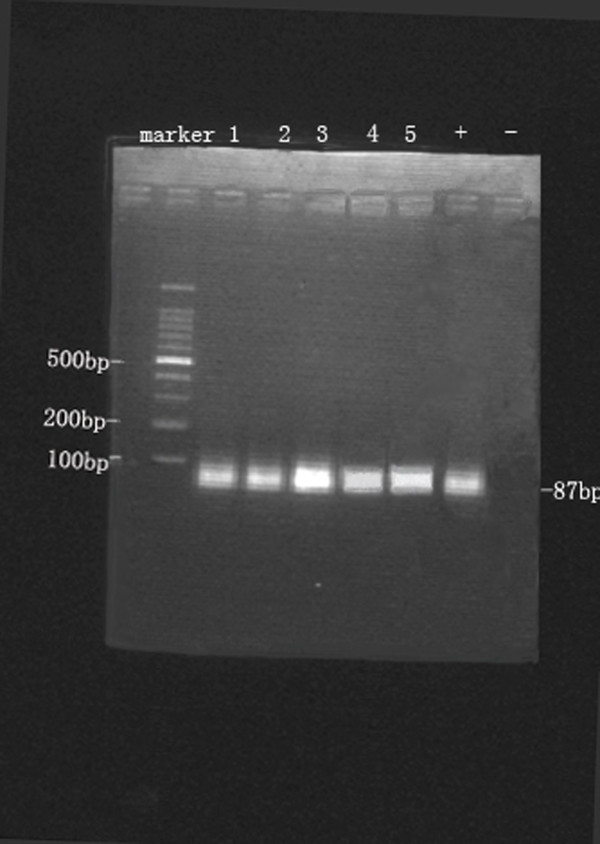
** *SYT-SSX* gene fusion was found in all five cases by RT-PCR.** The amplified fragments were identified by their size (*87* bp) on DNA electrophoresis using *1.5%* agarose gel. (−, negative control; +, positive control).

We performed the molecular genetic examination by a reverse-transcriptase polymerase chain reaction assay (RT-PCR) that detects (the paraffin blocks, formalin-fixed tissue) the *SYT–SSX* chimeric RNA transcript resulting from *t(X; 18)*, the characteristic phenotype of SS. The primers chosen detected both of the *SSX1* and *SSX2* partners, and the target sequence was 87 base pairs (bp) [3]. The presence of an *SYT–SSX* gene fusion was demonstrated from the paraffin blocks of all five cases by RT-PCR. The amplified fragments of the positive control and our cases were identified by their size (87 bp) on DNA electrophoresis using 1.5% agarose gel (Figure 6). Both pathologic and molecular examination confirmed the diagnosis of PPSS of the five cases.

Postoperative complications were found in three patients: two cases appeared with temporary neuropathies of the marginal mandibular branch of the facial nerve and one patient developed problems with malocclusion.

All five patients received postoperative radiotherapy (*50* to *70* Gy), and two patients with pulmonary metastasis received additional chemotherapy, including doxorubicin (*50* mg/m^2^) and ifosfamide (*7.5* g/m^2^).

At follow-up in May 2012, three patients had a tumor less than *5* cm: two patients were alive without a tumor for *54* and *57* months after surgery, respectively; and one patient with pulmonary metastasis received seven periods of chemotherapy and was still alive with a tumor for *78* months. Two patients with a tumor size larger than *5* cm died of the disease *26* and *37* months after the operation, respectively, because of local recurrence and multiple pulmonary metastatic foci.

Clinicopathological features of the five cases of PPSS are shown in Table 1.

**Table 1:** Clinicopathologic features of five cases of PPSS

**Case**	**Age**	**Tumor size**	**LN**	**Subtype**	**Treatment**	**Outcome**	**Survival**
*1*	*28*	*3.5 × 2.2 cm2*	N	monophasic	Excision + rad + chemo	ATPM	*78* m
*2*	*41*	*2.9 × 2.7 cm2*	N	biphasic	Excision + rad	ANED	*57* m
*3*	*22*	*4.0 × 3.1 cm2*	*0/*8	biphasic	Excision + rad	ANED	*53* m
*4*	*31*	*5.2 × 4.9 cm2*	*0/21*	biphasic	Excision + rad + chemo	DOR	*26* m
*5*	*37*	*6.6 × 5.1 cm2*	N	monophasic	Excision + rad	DOPM	*37* m

*ANED* alive with no evidence of disease, *ATPM* alive with pulmonary metastasis, *DOPM* died of pulmonary metastasis, *DOR* died of tumorous recurrence, *LN* indicates lymph node metastases, *m* months, *N* no check.

## Discussion

Neoplasms arising from the PPS are uncommon and account for only *0.5%* of all tumors that occur in the head and neck region. Reports in the literature indicate that *70* to *80%* of the parapharyngeal tumors are benign and *20* to *30%* are malignant [1]. Benign neurogenic and salivary gland neoplasms, such as pleomorphic adenoma, are the most common tumors. SS of the head and neck region are rare with less than *150* cases reported in the international literature, and PPSS is an extremely rare variant with scattered reports. Research reveals that SS originates from primitive mesenchyma with no synovial association in the PPS [4]. In the current study, we report five additional cases.

There are no specific features of PPSS in the clinical manifestations and the imaging modalities. Patients usually present a painless slow growing mass during their third and fourth decade of life with associated compressive or infiltrative symptom of surrounding structures, and males tend to be affected more often than females [5]. Consistent with the literature, our five patients were all men and their median age was *35* years (range *22* to *41* years). Four of the five patients presented a painless mass, and two of the five patients appeared dysphagic.

Microscopically, the classic forms of the SS have biphasic and monophasic patterns. When the biphasic pattern (epithelial and spindle) is observed, a definite diagnosis of SS may be possible. Monophasic fibrous SS, which is the most common subtype of SS, is very challenging to diagnose on histology. Therefore, immunohistological staining was significant in the differential diagnosis of SS from other sarcomas [6]. The tumor cells were positive for CD99, bcl-2 and focally reactive for CK, EMA in all five cases. S-100 protein was focally positive in one case. The nonreactivity of the tumor for SMA, desmin, CD34 and CD117 in our five cases argues against the diagnoses of leiomyosarcoma, rhabdomyosarcoma, solitary fibrous tumor and gastrointestinal stromal tumor [7]. Although malignant peripheral nerve sheath tumor is another sarcoma that usually occurs in the PPS, this tumor’s cells tends to show much greater atypia and S-100 protein immunohistochemical reactivity is more frequent than that seen in SS. In contrast, the CK positivity is more limited than that of SS.

The definitive diagnosis of SS should be made by molecular analysis, even more when the tumor arises in an unusual location, such as the PPS. Ninety percent of head and neck SS contains a specific translocation between chromosome X and 18, *t(x; 18)(p11.2;q11.2)*[2], that result in the presence of an *SYT-SSX1SYT-SSX2* and rarely *SYT–SSX4* gene fusion. The studies we performed demonstrated the presence of *SYT–SSX* (including *SYT-SSX1* and/or *SYT-SSX2*) fusion transcript in all five cases. Both pathologic and cytogenetic examination confirmed the diagnosis of PPSS. It was also demonstrated that RT-PCR with primers using RNA extracted from the paraffin blocks was feasible, as previously described [3].

Because of the lack of an established management protocol, the treatment of SS of the head and neck likely varies widely at different institutions. The therapeutic regimen should include surgical extirpation of the tumor followed by radiationtherapy when appropriate, with or without adjuvant chemotherapy. Limited excision is associated with a high incidence of local recurrence (*60* to *90%*) within two years of the original surgery [8], so wide local excision to ensure negative margins is the most important aspect of the treatment. Lymphatic spread is not a feature of the natural course of this tumor, and there is no established indication for a prophylactic neck dissection [9]. Use of adjuvant radiation therapy decreases the local recurrence rate [10]. The research by Al-Hussaini indicates that patients with localized SS have a good chance of cure with surgery and radiation therapy [11]. A single chemotherapy protocol has not yet been proven most effective in treating SS, but two agents, doxorubicin and ifosfamide, have demonstrated meaningful activity in the treatment of soft tissue sarcomas [12,13]. So according to the highmetastatic rate of these tumors, most authors recommend adjuvant chemotherapy as well as postoperative radiation therapy [2]. In this report, regional or total neck dissection was performed on two patients, and no lymph node metastases were found. All five patients were treated with surgical extirpation of the tumor and postoperative radiotherapy, and *60%* (three of five) of the patients were alive for more than four years. Two patients with pulmonary metastasis received additional chemotherapy. One patient remained disease stable, and in the other disease progressed and he died of the disease.

Combined modality therapy of this aggressive tumor yields better results; however, a five-year survival rate for these patients is poor and ranges from *25%* to *55%*[14]. Local or distant failure is seen in approximately *80%* of cases, and lung metastasis is the usual cause of death in patients with SS of the head and neck [5,15]. Penel et al. reported that poor prognostic factors of this tumor include initial metastasis or the presence of lymphadenopathy, absence of surgery and number of surgical procedures [16]. Other studies have shown that tumor size, histological type, status of surgical margins and anatomical location also play important roles in predicting local recurrence [17]. The research by Harb WJ et al. indicates that higher disease-specific and overall survival rates were associated with upper aero digestive tract location, tumors of ≤*5* cm, and tumors that did not extend into the bone [18]. Our follow-up date showed the prognosis of three patients with tumor size <*5* cm was better than the other two patients with tumor size >*5* cm. Because of the rarity of cases, we cannot draw any conclusion by statistical analysis.

## Conclusions

We describe five rare cases of PPSS. Accurate diagnosis depends on morphologic and immunohistochemical examination and proper molecular analysis. Like other sarcomas, definite diagnosis and tumor radical surgical resection at an early stage remain the mainstay of therapy. Adjuvant therapy, especially radiation, has some beneficial effect on the overall survival. We have shown that the prognosis of PPSS is better in those patients with tumor size less than 5 cm than in the patients with larger tumors.

## Consent

Written informed consent was obtained from the patients and their agents for publication of this case report and any accompanying images. A copy of the written consent is available for review by the Editor-in-Chief of this journal.

## Abbreviations

PPS, Parapharyngeal space; SS, Synovial sarcoma; PPSS, Primary parapharyngeal synovial sarcoma; FNA, Fine needle aspiration biopsy; RT-PCR, Reverse-transcriptase polymerase chain reaction assay; bp, Base pairs.

## Competing interests

The authors declare that they have no competing interests.

## Authors’ contributions

MZ, JL, KJW and JBS participated in drafting the manuscript and conducted critical reviews. JL carried out the histopathological evaluation and reviewed the pathology. All authors read and approved the final manuscript.
